# Does basic medical insurance promote public health? Evidence from China family panel study

**DOI:** 10.3389/fpubh.2023.1269277

**Published:** 2023-12-14

**Authors:** Fuchong Liao, Wenxiu Hu, Chun Zhang

**Affiliations:** ^1^Department of Public Administration, Central South University, Changsha, China; ^2^Centre of Population and Development Policy Studies, Fudan University, Shanghai, Shanghai, China; ^3^School of Business, Central South University, Changsha, China

**Keywords:** medical insurance, public health, health capital, sustainable livelihood, livelihood capital, common prosperity

## Abstract

**Background:**

To promote common prosperity, China government has devoted much financial resources to the basic medical insurance system, it is of great significance to improve the health level of the insured groups to prevent them from returning to poverty due to illness. Whether or not the basic medical insurance can improve health status is an important policy issue after China has win the absolute poverty alleviation movement.

**Methods:**

Based on the data of China Family Panel Studies this paper constructs a strong balanced panel data with two levels information, which including variables from family level and personal level. This paper uses the panel data fixed effect model and propensity score matching model to analysis.

**Results:**

This paper finds that after controlling the family and personal confounding variables, the basic medical insurance has positive effect toward health status. With propensity score matching model, this paper finds that there is causality between basic medical insurance and public health.

**Conclusion:**

Basic medical insurance has a significant health effect, that is, basic medical insurance has a significant positive impact on individual self-rated health. Participating in basic medical insurance can significantly improve the ability of families to face risk shocks, promote the accumulation of health capital in families, promote the diversification of livelihood strategies, and effectively prevent the occurrence of returning to poverty due to illness.

## 1 Introduction

Over the past few decades, governments and scholars have developed a great interest in directly engaging basic medical insurance reform and public health, known as universal basic medical insurance coverage in China (1–4). Accordingly, the construction of China’s medical insurance system continues to advance, and the policy goal of universal coverage of basic medical insurance is gradually advancing (3, 5). For the policy effect of basic medical insurance on public health, relevant studies have put forward many enlightening research conclusions. However, previous studies have two deficiencies:

First, from the perspective of the theory of common prosperity, there is a lack of research on basic medical insurance to alleviate relative poverty. After winning the battle against poverty, the main problems of poverty governance are relative poverty and common prosperity (6, 7). How to consolidate and expand the achievements of poverty alleviation through social security policies including basic medical insurance is a very important policy issue. Most of the existing studies on the poverty reduction effect of basic medical insurance are carried out in the context of absolute poverty standards and poverty alleviation policies (8, 9). There is a lack of attention to policy issues such as how to govern relative poverty and what role basic medical insurance can play after China wins the battle to fight poverty, which needs to be further strengthened.

Second, the existing research ignored the impact of family structural factors on public health to a certain extent. Whether to participate in medical insurance is an individual level behavior. Some studies only discuss the public health effect of basic medical insurance at the individual level, ignoring the impact of family structural factors (4, 10). Although some relevant studies have controlled structural factors such as the number of family population (11), family dependency ratio and the characteristics of family heads when discussing the public health effect of basic medical insurance (1, 4), they lack a consistent analysis framework, and the theoretical basis of model construction needs to be strengthened.

Therefore, under the consistent framework of sustainable livelihoods, this paper uses the panel data of the national large sample household survey to explore the impact of basic medical insurance on public health. And the research question of this paper is:

*Does basic medical insurance promote public health?*

From the perspective of theoretical contribution, by incorporating family structural factors such as livelihood capital and livelihood strategies into the discussion, this paper contributes to an emerging livelihood capital framework of literature which examines relationship of basic medical insurance and public health. Besides, from the perspective of policy implications, by focusing on the impact of basic medical insurance on health status, this paper puts forward corresponding policy suggestions on basic medical insurance reform.

In the remaining parts of this paper, we first develop hypotheses based on the sustainable livelihood framework and a review of relevant literature. We then introduce our research design, including the statistical method, data and variables. After presenting the results, we discuss the theoretical and practical implications.

## 2 Literature and hypothesis

### 2.1 Medical insurance and public health

The existing researches mainly put forward the theoretical mechanism of basic medical insurance improving the public health from two aspects of direct and indirect effects:

First, from the perspective of direct effect, the reimbursement system of basic medical insurance, to a certain extent, improves the enthusiasm of the insured for treatment, partially eliminates the worries of going to the hospital for medical examination (2), and prevents further damage to health due to the delay of examination and treatment time. In China’s rural areas, there is a relatively common situation that the disease is not checked and treated in time, resulting in a minor illness dragged into a major disease (5). Based on the study of the new rural cooperative medical system in China, it is found that when the insured farmers have discomfort and disease risk, their probability of going to the hospital for examination and treatment is significantly higher than that of uninsured farmers (3, 9).

Second, from the perspective of indirect effects, the basic medical insurance can cover part of the treatment costs, reduce the squeeze on other family consumption, and is conducive to the long-term sustainable livelihood development of families (12). The development of China’s basic medical insurance system has provided poor families with stronger confidence to carry out small-scale investment and large-scale production activities (13). Due to the existence of the basic medical insurance system, poor households have certain psychological expectations and can get a certain proportion of reimbursement and compensation of basic medical insurance in the expenses of disease treatment (9). It is based on this point that the study found that in the areas with a high participation rate of new rural cooperative medical system, poor households are more active in diversified livelihood investment (1). In fact, an important part of the health poverty alleviation project is to achieve the goal of full coverage of basic medical insurance for poor households through financial investment (14). The central budget carried out special transfer payments in concentrated and contiguous special poor areas, and promoted the realization of the basic medical care of poor households (15). Objectively speaking, the health poverty alleviation project significantly improves the health status and human capital accumulation of poor families by improving the labor capacity of poor people and reducing the squeeze of medical expenses on other family expenditures (16).

However, the extant literature on this topic continues to suffer from two general limitations: (1) Most literature lacks attention to the household livelihood structure, and some studies have overlooked key variables such as livelihood capital and livelihood strategies. Livelihood capital and livelihood strategies are important factors that affect the outcome of household livelihoods. Household income, consumption, welfare status, and incidence of poverty are all influenced by livelihood capital and livelihood strategies. The existing research mainly explores the poverty reduction effect of basic medical insurance from two perspectives: reducing poverty vulnerability through basic medical insurance. (2) In terms of variable control, relevant empirical studies have focused on individual level related variables, such as gender, age, marital status, whether to smoke, whether to drink alcohol, etc. However, attention to family structural factors needs to be strengthened, especially the lack of attention to family livelihood capital and strategies. Therefore, we need theoretical insights and empirical evidence to test the relationship between basic medical insurance and public health.

This paper uses nationally representative large sample household survey data to explore the health effect of basic medical insurance within the theoretical framework of sustainable livelihoods. To some extent, our research has expanded the boundaries of the use of sustainable livelihood theory, enhanced the understanding of medical health effect, this paper has made marginal theory contribution of the public health.

### 2.2 Sustainable livelihood framework

the sustainable livelihood framework has been widely discussed in the development discourse variously as an analytical framework, a development objective and even an approach to policy decision-making (2, 17, 18). In this paper, the sustainable livelihood framework is treated as a framework for analysis the relationship between basic medical insurance and public health. The sustainable livelihood framework systematically describes the various links and relationships of family livelihood activities (19–21), including the following two elements: (1) Livelihood capital. Livelihood capital is the basis for families to carry out livelihood activities, including five types of material capital, natural capital, financial capital, human capital and social capital (18, 22). Livelihood capital is dynamic, and different types of livelihood capital can be converted to each other. (2) Livelihood results. The livelihood result is the last link of family livelihood (23), which is embodied in the change of family income, the change of family consumption expenditure and the improvement of life quality and welfare. It should be noted that the livelihood results of families in a specific cycle will have a very significant impact on livelihood capital, and this process is named livelihood feedback. Specifically, positive livelihood results such as income consumption increase and welfare improvement will form positive feedback on livelihood capital, which is conducive to the further accumulation of livelihood capital (19, 24). In this case, a virtuous cycle between livelihood capital and livelihood results is formed (20, 21). The virtuous cycle and feedback of family livelihood activities is of great significance for poverty alleviation and improving the quality of life (25). In addition, vulnerability background is an important variable affecting family livelihood activities. In the areas with fragile ecological environment and insufficient resource bearing capacity, residents’ livelihood activities will be greatly affected. On the contrary, residents living in resource rich and environment-friendly areas are more likely to form a virtuous cycle in their livelihood activities.

**Figure 1:**
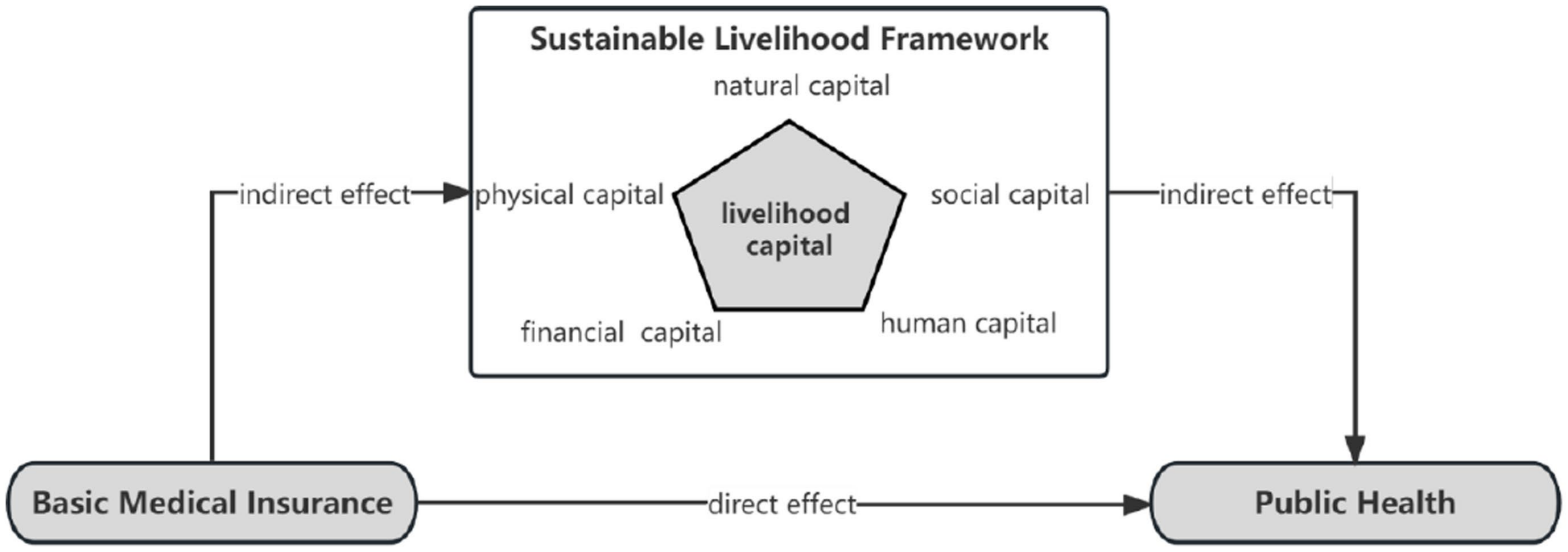
Research model.

Based on theorical analysis, we propose hypothesis (Figure 1):

*H1*: basic medical insurance is positively related to public health.

*H2*: livelihood capital is positively related to public health.

*H3*: the positive relation between basic medical insurance and public health is causality.

## 3 Methods

### 3.1 Data sources

This study uses household-level and personal-level survey data from China Family Panel Studies, which performed by Peking University. China Family Panel Studies is a nationally representative social tracking survey data covering 25 provinces in mainland China, it started from 2010, and until now there are six waves from 2010 to 2020. The CFPS adopts computer-aided survey technology, with the aim of collecting data from three levels: village, family, and individual, to reflect the changes in China’s society, population, and family. The survey database covers village sample, family sample, adult sample and child sample. In addition to the conventional demography variables, the survey also includes family economic and asset status, employment and income of family members, health status, population migration, public services and other variables. As for the data time, the 2010 is baseline survey, and the 2012 is the first tracking survey, the 2014 is the second tracking survey, the 2016 is the third tracking survey, the 2018 is the fourth tracking survey, and the 2020 is the fifth tracking survey. That is to say, the 2020 CFPS data is the latest data until now. This survey covers a wide range of household and personal economic and demographic variables, and it has been widely used to study China’s social security and public healthy questions.

In processing data, in order to avoid the influence of outliers, we winsorised these variables at 1st and 99th percentiles. Therefore, based on the data of China Family Panel Studies, this paper constructs a large sample balanced panel data. The panel database includes more than 15,000 samples of tracking adults and more than 6,000 tracking families, covering information at both individual and family levels.

### 3.2 Variable measurement

In terms of variable measurement, basic medical insurance is the main independent variable of this paper, which aims to explore how basic medical insurance affects the public health. Different types of basic medical insurance have different protection effects, and their effects on family income and welfare are also quite different (26–29). The dependent variable in this paper is health status, and the control variable includes related variables at the individual level and family level. For the definition of variables and measurement indicators in this paper, see Table 1 for details.

**Table 1:** Variables measurements and variables code.

Variables	Variables description	Variables measurements	Variables code
Dependent variable	Health status	Self-assessment of health	1–5, 1 = very unhealthy 5 = very healthy
Independent variables	Medical insurance	Dummy variable, whether or not have medical insurance	1 = have medical insurance, 0 = do not have medical insurance
	Type of medical insurance	Category variables, with do not have medical insurance as comparison	Basic medical insurance for urban employees, basic medical insurance for urban residents, and new rural cooperative medical system
Personal level control variables-	Gender	Dummy variable, male and female	Male = 1, female = 0
	Age	Age in the survey year	Actual age
	Marriage	Marriage status	1 = in marriage, 0 = unmarried
	Education	Education year	Total school education years
	Smoker	Smoking or not	1 = smoking, 0 = non-smoking
Family level control variables	Natural capital	Market value of family owned land (yuan)	Actual market value of land
	Physical capital	Residential housing status	1–5,1 = bad crowding, 5 = spacious and comfortable
	Financial capital	Market value of household financial assets (yuan)	Actual market value of financial assets
	Human capital	Average length of schooling of family members (years)	Average school education years
	Social capital	Can I borrow money from relatives and friends	1 = yes 0 = no
	Family income	Total family income with log	Yuan

Data from China Family Panel Studies (2010–2020).

The independent variable is the type of basic medical insurance. This variable is a category variable. In the original database there are five options for medical insurance, namely “public medical care,” “basic medical insurance for urban employees,” “basic medical insurance for urban residents,” “supplementary medical insurance” and “new rural cooperative medical care.” In China’s current medical security system, there are mainly three types: the basic medical insurance for urban employees, the basic medical insurance for urban residents and the new rural cooperative medical system. Others, such as supplementary medical insurance and public medical care, are not the main form of China’s basic medical security system, but an important supplement to the medical insurance system. In addition, because the overall coverage rate of public medical care and supplementary insurance in China is low, and the sample size involved in the database is small, this paper mainly retains the following three types of medical insurance, namely, the basic medical insurance for urban employees, the basic medical insurance for urban residents and the new rural cooperative medical system.

The dependent variable is individual self-rated health. There are three main ways to measure “health status” in existing studies: neonatal mortality, health scale and self-rated health. First of all, from the perspective of measurement sensitivity, the self-rated health index has the highest sensitivity, which is actually the health scale, and the measurement index of neonatal mortality has the lowest sensitivity. The reason for the low sensitivity of mortality indicators is that the reduction of barriers such as the reduction of morbidity may be ignored. The core of health scale measurement is the measurement of limb function, which is generally used for the older adults. The advantage of self-rated health mainly reflects the respondents’ comprehensive judgment on their own physical condition, which is relatively comprehensive. Secondly, from the perspective of objectivity, the index measurement of neonatal mortality is the most objective, the health scale ranks second, and the measurement method of self-rated health is the most subjective. Based on the above comparison of health status measurement indicators, it can be found that self-rated health is also an important measure of individual health level. Compared with the use of objective disease indicators to measure the health level, the individual’s comprehensive score for their own health level is based on the objective health status to a certain extent (30–32). Self-rated health can integrate multiple factors and can be used as a proxy variable of health status.

The control variables include livelihood capital, based on sustainable livelihood framework. This paper uses five different ways to measure the livelihood capital: (1) Natural capital is land and its output, including cultivated land, forest land, wildlife and food, biodiversity, etc. Because the value of land is different in different regions and natural conditions, and the land area does not well reflect the attribute of land as a means of production, this paper mainly selects the market value of land as the measurement of family natural capital. (2) In terms of financial capital, this paper uses the market value of household financial assets to measure. The financial assets here include family savings, stocks, options, funds, financial products and other types. Using market value to measure can comprehensively cover the basic situation of different types of financial assets, which is convenient for summary calculation and comparison. (3) In terms of human capital, this paper uses the respondents’ maximum years of education to measure. Human capital mainly includes the family labor force, the education level of family members and the health of family members. Specifically, it includes the size of family population, the number of family labor force and the highest education level of family members. Therefore, in discussing the related issues of family sustainable livelihood, education level has received more attention, and education level has also been directly used as the main indicator of family human capital in some studies. (4) In terms of physical capital, this paper uses the residential housing status as measurement, range from 1 to 5. And the 1 means bad crowding, with 5 means spacious and comfortable. (5) In terms of social capital, this paper uses the ability to borrow money from relatives and friends to measure. The original title article “how much (yuan) did your family borrow from relatives/friends in the whole year of last year”? This article has carried out coding conversion, 1 means you can borrow money, 0 means you cannot borrow money. There is a problem here. Can we borrow money and measure the social capital of families? The author believes that the answer is yes. Social capital is mainly associated with interpersonal networks. The main measurement indicators include the number of relatives and friends who pay New Year greetings during the Spring Festival, the number of relatives and friends who can borrow money, the frequency of family and friends’ dinners, whether they participate in industry professional associations, and whether there are veterans and village cadres among family members.

### 3.3 Empirical estimation

In this paper, we will use panel data fixed effect model and propensity value matching to explore the impact of basic medical insurance on health status. Based on the above analysis, it can be found that the Chinese household tracking survey is a short panel data with strong balance, which can be analyzed by using the panel data model. Panel data regression analysis can explore the influence of a variety of factors on dependent variables, which is suitable for this paper.

Propensity score matching (PSM) is one of the important methods for causal inference. From the perspective of the basic principle of propensity value matching, it is actually to statistically construct an experimental group and a control group through the survey data, construct a counterfactual framework based on the observation data by matching the covariates, and then identify the causal relationship between the pair of core policy independent variables and the result variable. Medical insured and uninsured are a typical binary dummy variable, which can also be understood as a special policy stimulus. Therefore, the propensity score matching method is suitable for discussing the impact of medical insurance on relative poverty.

In the theoretical framework of sustainable livelihoods, health status can be an aspect of livelihood outcomes. The livelihood result is the result of households’ production and operation activities through specific livelihood strategies based on the livelihood capital stock. In the theoretical framework of sustainable livelihoods, livelihood capital is also a “stock,” which will have a very significant impact on livelihood strategies and outcomes. In this sense, the health demand model proposed by Grossman (33) is similar to the sustainable livelihood framework. Therefore, this paper integrates the above two theoretical frameworks and proposes an empirical equation to explore the impact of medical insurance on residents’ health


$$\mathrm{Healt}h_{\mathrm{it}}=\beta_{0}+\beta_{1}\;\mathrm{Medcar}e_{\mathrm{it}}+\beta_{2}\;X_{\mathrm{it}}+\lambda_{t}+\eta_{t}+\varepsilon_{\mathrm{it}}$$

$\mathrm{Healt}h_{\mathrm{it}}$ refers to the self-rated health level of individual *i* at time *t*, $\mathrm{Medcar}e_{\mathrm{it}}$ _ It refers to the medical insurance coverage of individual *i* at time *t*. This paper uses two ways to define and divide medical insurance: the first level, whether to participate in insurance, uses the dummy variable coding method of 0 and 1. The second level is the type of insurance. According to the previous review, the basic medical insurance system consists of the basic medical insurance for urban employees, the basic medical insurance for urban residents and the new rural cooperative medical system. In addition, there are other forms such as supplementary medical insurance and commercial medical insurance. For different types, this paper compares and analyzes different medical insurance categories through the coding method of dummy variables, and $\beta_{1}$ is the main coefficient of this paper. $X_{\mathrm{it}}$ refers to the individual characteristics and family characteristics of individual *i* at time *t*, that is, the control variables at individual level and family level. $\lambda_{t}$ is the time fixed effect, $\eta_{t}$ is the provincial fixed effect, which can solve the missing variables that do not change with time at the provincial level, $\varepsilon_{\mathrm{it}}$ is the error term that changes with time.

## 4 Results and discussion

### 4.1 Spearman correlation analysis

this paper analyzes the correlation of independent variables, dependent variables and control variables in the panel database. See Table 2 for the correlation coefficient table of each variable at the individual level.

**Table 2:** Spearman correlation analysis.

	Health	Insurance	Expenses	Out-of-pocket	Insurance type	Smoker
Health	1					
Insurance	0.03*	1				
Expenses	0.08*	-0.02*	1			
Out-of-pocket	0.09*	−0.03*	0.64*	1		
Insurance type	0.03*	1.00*	−0.11*	−0.05*	1	
Smoker	−0.04*	−0.015	0.06*	0.05*	−0.02*	1

*p < 0.05; **p < 0.01; ***p < 0.001.

The results of correlation analysis showed that the correlation coefficient between whether to participate in insurance and self-rated health was 0.03, the correlation coefficient with medical expenses was−0.02, The above correlation coefficients were significant at a 95% confidence level. The correlation coefficient between whether to participate in insurance and whether to smoke is-0.01, but it is not significant. The types of insurance participation are ordered variables, including specific types such as basic medical insurance for urban employees, basic medical insurance for urban residents, new rural cooperative medical insurance, supplementary medical insurance, and commercial medical insurance. The correlation coefficient between the type of insurance and self-rated health is 0.02, the correlation coefficient with medical expenses is-0.02, and the correlation coefficient with self-paid medical expenses is -0.01.

### 4.2 Fixed effect model

Based on the balanced Panel data of the China Family Panel Studies, this paper constructs a panel data fixed effect model as a benchmark. See Table 3 for the analysis results of the benchmark model.

**Table 3:** The health effects of medical insurance (fixed effect model).

	Dependent variable: public health			
	Model 1	Model 2	Model 3	Model 4
Medical insurance	0.328***	0.345***	0.247***	0.258***
	(0.018)	(0.021)	(0.017)	(0.019)
Control variables (Individual level)				
Age		0.027***		0.028***
		(0.000)		(0.001)
Gender		0.011		0.178
		(0.219)		(0.253)
Marriage		−0.192***		−0.149***
		(0.037)		(0.038)
Education		0.016*		0.012
		(0.006)		(0.008)
Smoking		−0.262***		−0.253***
		(0.026)		(0.021)
Control variables (Family Level)				
Physical capital			0.013	0.018**
			(0.007)	(0.006)
Natural capital			0.001	0.000
			(0.000)	(0.000)
Financial capital			0.013***	0.014***
			(0.001)	(0.002)
Social capital			0.074**	0.151***
			(0.018)	(0.020)
Human capital			0.027***	0.020***
			(0.002)	(0.003)
Family income (log)			0.057***	0.049***
			(0.008)	(0.006)
Time fixed effect	No	Yes	Yes	Yes
Provincial fixed effect	No	Yes	Yes	Yes
				
Constant	2.604***	0.991***	2.147***	0.549**
	(0.016)	(0.139)	(0.042)	(0.108)
N	59,574	42,997	43,125	34,017
adj. *R*^2^	0.287	0.278	0.335	0.284
F-statistic	347.339***	765.215***	183.047***	453.082***

1. The brackets indicate robust standard error; 2. *p < 0.05; **p < 0.01; ***p < 0.001; 3. Missing value have been deleted.

Model 1 is a null model, with medical insurance participation as the independent variable and self-assessed health level as the dependent variable. The zero model does not include control variables, nor does it control for time fixed effects and province fixed effects. Research has found that compared to the group without medical insurance, respondents who participate in medical insurance have a better self-assessment of their health level, with an estimated coefficient of 0.328, which is significant at a 99.9% confidence level. The sample size of Model 1 is 59,574, adjusted *R*^2^ is 28.7%, F-statistic is 347.339, and value of p is less than 0.000.

Model 2 adds individual level control variables based on Model 1, including age, gender, education level, and smoking behavior, while controlling for time fixed effects and province fixed effects. The model estimation results indicate that participating in basic medical insurance has a significant health effect, while controlling for individual characteristics and fixed effects of time provinces. The estimated coefficient for participating in medical insurance is 0.345, which is significant at a 99.9% confidence level, indicating that compared to the group without medical insurance, the group participating in medical insurance has a higher level of health. The control of time fixed effects is achieved by adding dummy variables of the survey year to the model estimation, while the province fixed effect is achieved by adding dummy variables of the survey province code to the model. The sample size of Model 2 is 42,997, with an adjusted R^2^ of 27.8% and an F-statistic of 765.215, with a value of p less than 0.000.

Model 3 further adds family level control variables on the basis of model 1 and referring to the relevant research of the theoretical framework of sustainable livelihoods (22, 34), including livelihood capital variables in five dimensions of physical capital, natural capital, human capital, financial capital and social capital (19), as well as relevant measurements of livelihood strategies, and controls the time fixed effect and provincial fixed effect. The estimation results of the quantitative model show that after controlling for household level livelihood capital and livelihood strategy variables, medical insurance still has a significant health effect. This indicates that compared with the group who did not participate in medical insurance, the group participating in medical insurance has better health conditions and has a significant health effect. In terms of controlling variables in the family dimension, livelihood capital has a significant positive impact on health status (10, 18). The positive impact of diversification of livelihood strategies on household livelihood outcomes and health status is relatively consistent with existing research findings on diversification of livelihood strategies. The sample size of Model 3 is 43,125, with an adjusted R^2^ of 33.5% and an F-statistic of 183.047, with a value of p less than 0.000.

Model 4 is full model, based on model 1, control variables at the individual level, family level, time fixed effect and provincial fixed effect are added. The model estimation results limit that after controlling for individual level, family level, and time province fixed effects, medical insurance still has a significant positive impact on health status. Specifically, the estimated coefficient for participating in medical insurance is 0.258, which is significant at a 99.9% confidence level, indicating that compared to the group who did not participate in medical insurance, the group participating in medical insurance has better health conditions and has a significant health effect. The sample size of model 4 is 34,071, and the main reason for sample missing is that the Saturated model includes many control variables, and some samples are lost after deleting the missing values. The adjusted *R*^2^ of Model 4 is 28.4%, the F-statistic is 453.082, and the value of p is less than 0.000.

In summary, medical insurance still has significant health effects even when controlling for individual level variables, family level variables, time fixed effects, and provincial fixed effects (25). In this sense, promoting full coverage of basic medical insurance for impoverished households can help prevent and control the occurrence of poverty caused by illness and returning to poverty due to illness (2), which is consistent with existing research findings (35).

The estimation coefficient calculated by the above panel data fixed effect models are still the regression analysis in essence, so it is necessary to further explore the causal relationship between medical insurance and health status. The following paper uses the Causal inference method of propensity score matching to analyze the causal link between medical insurance and health status in the framework of consistency of sustainable livelihoods (20, 21).

### 4.3 Propensity score matching

In this paper, causal inference is carried out according to the following steps:

First, calculate the propensity score. Generally speaking, the method of sample matching is to predict the probability of accepting policy stimulus through statistical model, that is, the score of propensity value. The research topic of this paper is the probability of participating in basic medical insurance. Under the consistent framework of sustainable livelihood theory, this paper controls the relevant factors at the individual level and the variables such as livelihood capital and livelihood strategies at the family level, and predicts the propensity score of participating in medical insurance through the commonly used logit model.

Second, the sample matching and covariate deviation were compared. After obtaining the score of social groups’ tendency to participate in medical insurance in the above steps, the groups with similar scores can be matched according to specific methods. In a general sense, there are three commonly used sample matching methods: one is proximity matching, that is, according to a certain matching proportion, matching samples with similar scores but different independent variables. The second is radius matching, that is, select paired samples within a certain radius. The third is kernel function matching, which determines the matching range through a specific function. In this paper, three matching methods are used to obtain more accurate coefficient estimation. The proportion of adjacent matching is 1 to 4, that is, one uninsured individual matches four insured individuals. This matching proportion is consistent with the overall distribution of the sample, which can maximize the use of sample information. The default radius of 0.01 is used for radius matching.

Through the adjacent matching of the sample propensity score in the ratio of 1 to 4, it can be found that the covariate deviation is effectively reduced after matching, which is realized as a reference baseline closer to 0 in the coordinate in the figure. After matching, the covariate deviation becomes smaller, which theoretically means that the difference between the paired samples at the individual level and the family livelihood level is very small. Statistically, it can be considered that the only source of the difference in the health status of paired individuals is policy stimulus (36, 37). In this paper, policy stimulus is a binary dummy variable of whether to participate in insurance.

Third, the common support test. After comparing the covariate bias, it is necessary to further test the common support domain. After obtaining the propensity values of the research individuals in the above steps and comparing the covariate deviations, it can be found that the propensity values of some research objects in the database are too high or too low to find similar matching objects. It is difficult for respondents whose propensity scores are in the abnormal value range to find similar matching objects, which is also reflected in the large deviation at the covariate level after matching. Because of the existence of individuals with abnormal propensity score, it is necessary to deal with this part of the sample. The common support domain test can find the research objects with abnormal propensity value, and delete these objects in one-step analysis, in order to obtain more accurate causality coefficient.

The test results of the common support domain show that the experimental group and the control group have better matching effect after neighborhood matching, that is, the research objects who participate in medical insurance and the research objects who do not participate in medical insurance can be matched. After passing the common support domain test, it means that the matched samples only have the difference of whether to participate in medical insurance or not, but are highly similar at the individual level and the family livelihood level (20, 21). This means that the difference between the groups participating in medical insurance and the groups not participating in medical insurance in the survey sample is only the difference in medical insurance participation (38).

Finally, the causality coefficient is obtained. Through the above steps, this paper obtained the causality coefficient of the impact of basic medical insurance on the health status of the research object. The causality coefficient can be further divided into ATT, ATU and ate. The average treatment effect on the treated (ATT) refers to the policy effect of the experimental group, which is the core result of propensity score matching. This paper calculates the causality coefficient of three different matching methods and further reports the ATT coefficient.

This shows that in the research topic of health effects of medical insurance, after controlling relevant variables based on sustainable livelihood theory, the endogenous problem between medical insurance and health status does not pose a serious challenge, which further proves the robustness of the analysis results in this paper. Theoretically, the basic medical insurance for urban employees is compulsory (10, 18), and there is no self-selection problem of samples, that is, there is no self-selection bias. The basic medical insurance for urban residents and the new rural cooperative medical system are mainly voluntary, and the participation rate is high under the government’s incentive policies (35).

### 4.4 Discussion

This paper discusses the basic medical insurance system and the public health under the theoretical framework of sustainable livelihoods. The possible research contributions focus on theoretical promotion and quantitative causal identification.

From the perspective of theoretical promotion, this paper discusses the construction of basic medical insurance system and public health based on the theory of family sustainable livelihood, which expands the applicable boundary of sustainable livelihood theory to a certain extent. The theory of family sustainable livelihoods is an analytical framework that has been widely used in the related research of poverty governance. The existing research has made many beneficial extensions in the dimensions of livelihood capital and livelihood strategies, which has deepened the understanding of poverty governance and family livelihoods in the academic community (17, 19). The relevant research on the poverty reduction effect of basic medical insurance is mainly carried out under the standard of absolute poverty and the policy scenario of poverty alleviation (10, 18). Rich research results have been accumulated on the related issues of basic medical insurance to prevent poverty caused by illness and return to poverty due to illness. Poverty governance after 2020 is mainly relative poverty. It is a very important policy issue to explore how to reduce poverty from the perspective of family sustainable livelihood theory. Unfortunately, the research on the poverty reduction effect of basic medical insurance under the relative poverty standard needs to be strengthened (25), and there is a lack of consistent analysis framework in the relevant research. Based on the large sample panel data of China’s household tracking survey, this paper discusses the policy effect and impact mechanism of basic medical insurance on relative poverty under the consistency framework of sustainable livelihood theory, and puts forward the analysis framework of basic medical insurance to reduce relative poverty from the perspective of sustainable livelihood, which expands the use boundary of sustainable livelihood theory to a certain extent, It enhances the understanding of poverty reduction through basic medical insurance in relative poverty governance, and realizes the expansion in the marginal sense at the theoretical level.

From the perspective of quantitative causal identification, this paper constructs a nationally representative large sample of balanced panel data, establishes the causal link between basic medical insurance and relative poverty, and makes a certain marginal contribution in the level of quantitative causal identification. Based on the adult data, family data and village community data of the China Household tracking survey from 2010 to 2020, this paper constructs a large sample of balanced panel data with a time span of 10 years, and analyzes the health effect by using panel fixed effect model. At the further causal identification level, this paper constructed a statistical experimental group and a control group by using the method of propensity score matching, discussed the impact of the basic medical insurance system on the results of family livelihood, and established the causal link between the basic medical insurance participation and the relative poverty reduction. In terms of further impact mechanism analysis, this paper establishes the regulatory effect model of livelihood strategies on the basis of sustainable livelihood theory (20, 21), and puts forward the regulatory effect mechanism of diversified livelihood strategies to reduce poverty. It has made a marginal contribution to the causal inference of the basic medical insurance system and public health.

## 5 Conclusion

To promote common prosperity, we need to focus on solving the problem of relative poverty. Through the construction of the basic medical insurance system, it is of great significance to improve the health level of the insured groups to prevent them from returning to poverty due to illness. Based on the Chinese Family Panel Studies (CFPS), this paper constructs a large sample of balanced panel data covering individual information and family information. Under the consistent framework of sustainable livelihoods, this paper discusses the impact of basic medical insurance on health status through panel fixed effect model and propensity value matching method.

This article constructs panel data fixed effects models through the analysis of large sample data from Chinese Family Panel Studies. The study found that after controlling for individual level factors, family level factors, time fixed effects, and provincial fixed effects, the group participating in basic medical insurance had better health conditions compared to the group not participating in medical insurance, that is, basic medical insurance had a significant positive impact on self-rated health. The causal inference of propensity value matching found that there was a causal relationship between basic medical insurance and health status.

this study also provides applicable insights for basic medical insurance public administration practitioners: (1) improving financial investment in the basic medical insurance system and continue to promote the policy goal of universal coverage of basic medical care. Through the construction of econometric model and data analysis, this paper found that compared with the group who did not participate in the basic medical insurance, medical insurance can bring significant health effects and income effects, and significantly reduce the incidence of poverty in families. From this point of view, the realization of universal coverage of basic medical insurance is of great significance in preventing poverty caused by illness and returning to poverty due to illness. Participating in basic medical insurance can significantly improve the ability of families to face risk shocks, promote the accumulation of health capital in families, promote the diversification of livelihood strategies, and effectively prevent the occurrence of returning to poverty due to illness. By increasing the financial investment in basic medical insurance, continuing to promote the construction of the basic medical security system, and further promoting the universal coverage of the basic medical insurance system, it is of great significance to realize the greater goal of common prosperity. (2) coordinate and promote the construction of the medical security system, and form an institutional pattern in which the basic medical security system is the main body and various forms of medical security develop together. The macro medical security system includes basic medical insurance, medical assistance, supplementary medical insurance, commercial health insurance, charitable donations and mutual medical assistance. The basic medical insurance is the main body, and other forms of medical security are important supplements. In China’s medical insurance system, there are three main components: the main system is the basic medical insurance for urban employees and the basic medical insurance for urban and rural residents. The basic medical insurance system for urban employees mainly covers the population working in cities and towns. The fund account is composed of a financial pooling account and a personal account, in which the payment of the personal account is shared by employees and units. In addition, the supplementary system is commercial health insurance and other medical insurance. The supplementary medical security system is mainly funded by individuals, without financial subsidies. The supplementary medical insurance system is mainly aimed at the higher-level medical security needs of high-income people. Finally, the bottom line system is the urban and rural medical assistance system. The urban and rural medical assistance system aims at the impact of unexpected medical events on individuals and families, and aims to alleviate the urgent needs of the people in distress through donations and relief funds. To further play the poverty reduction effect of the medical insurance system, we need to use a variety of ways and means to estimate that conditional families can participate in various forms of supplementary medical insurance, improve the ability to resist risks and reduce the vulnerability of livelihoods in addition to the basic medical insurance system. Further form a collaborative governance pattern with the basic medical insurance system as the main body and the common development of various forms of medical insurance.

## Data availability statement

The original contributions presented in the study are included in the article/Supplementary material, further inquiries can be directed to the corresponding author.

## Author contributions

FL: Conceptualization, Formal analysis, Writing – original draft, Writing – review & editing. WH: Funding acquisition, Resources, Visualization, Writing – original draft. CZ: Conceptualization, Data curation, Methodology, Project administration, Writing – review & editing.
